# MALDI-TOF MS-based analysis of dried seed proteins immobilized on filter paper

**DOI:** 10.1093/biomethods/bpz007

**Published:** 2019-06-20

**Authors:** Michael A Reeve, Kathryn M Pollard

**Affiliations:** CABI, Bakeham Lane, Egham, Surrey, UK

**Keywords:** Dry protein storage, Himalayan balsam, Matrix-assisted laser-desorption and ionization time-of-flight mass spectroscopy, Plant-biotype discrimination, Seed-protein analysis

## Abstract

Matrix-assisted laser-desorption and ionization time-of-flight mass spectroscopy (MALDI-TOF MS) is commonly used for the characterization of protein-containing biological samples. For this, we have previously developed sample-preparation methods that can be used for discrimination between *Impatiens* species and also between regional biotypes of Himalayan balsam (*Impatiens glandulifera*), initially using leaf samples and, more recently, using seed material. In the current article, we have developed a further MALDI-TOF MS-based method that can be used with seeds that uses only simple equipment and minimally hazardous reagents prior to storing and/or shipping dried seed proteins immobilized on filter paper for MALDI-TOF MS analysis. We have investigated *I. glandulifera* regional-biotype seeds originating from four different sites within the UK for which the parent plants differ in their susceptibility to the biological control agent *Puccinia komarovii* var. *glanduliferae*. Using a combination of time-course comparisons and principal-component analysis, we have demonstrated good MALDI-TOF MS spectral conservation, even after storage for 1 month at 35°C, of dried seed-protein samples immobilized on filter paper. This method may provide a further useful tool for the matching of biological control agents optimally to susceptible (regional) target-plant biotypes, and for seed characterization and/or identification in general.

## Introduction

Originating from the Himalayas, the annual plant *Impatiens glandulifera* (Balsaminaceae), known commonly as Himalayan balsam, was introduced to the UK as a garden ornamental through Kew Gardens in 1839 [1]. Since its initial introduction, *I. glandulifera* has naturalized, spreading throughout the UK to become one of the most-prevalent invasive species, forming monocultures at high plant density which reduce biodiversity and which can have a detrimental impact on entire ecosystems [2–6]. In 2006, CABI responded to this threat by initiating a program of biological control against *I. glandulifera*, within which surveys were conducted to search for natural enemies within the plant’s native range [7]. Resulting from these surveys, a rust fungus (subsequently identified as *Puccinia komarovii* var. *glanduliferae* [8]) was selected for further assessment as a possible biological control agent. Criteria for selection included the rust’s prevalence and the high level of damage it is capable of inflicting on *I. glandulifera* in the field [9]. After extensive safety testing to ensure that the fungus was reliable, effective, and safe, posing no risk to UK biodiversity, a strain of the rust from India was approved for release in 2014 [9]. 

Initial releases of the Indian rust strain were conducted in England and Wales in 2015 and 2016, revealing that regional *I. glandulifera* biotypes can differ in their susceptibility to this particular biological control agent. Some regional biotypes are observed to be fully susceptible to the rust whilst others are seen to be resistant [10]. These observations, combined with those reported by Nagy and Korpelainen [11], who concluded that *I. glandulifera* has been introduced into the UK on multiple occasions from both India and Pakistan within the native range, suggest that there are multiple regional biotypes of *I. glandulifera* growing within the UK. In response to the above, a second strain of *Puccinia komarovii* var. *glanduliferae*, originating from Pakistan, was investigated. Initial assessments with this strain found that it can infect a significant number of regional biotypes that are resistant to the Indian rust strain. Permission to release this second strain was granted by the UK Government in January 2017 and, following assessments of regional-biotype susceptibility, the rust strain originating from Pakistan was released at UK field sites during 2017.

Our goal is to provide rapid and inexpensive methods for characterizing differences between plant regional biotypes that can be used to optimize the matching between a given biological control agent and susceptible target-plant biotypes. Of the available techniques, matrix-assisted laser-desorption and ionization time-of-flight mass spectrometry (MALDI-TOF MS) has the advantage of being both rapid and relatively inexpensive. In this technique, the MALDI soft ionization process [12] allows the desorption of proteins intact into the gas phase, carrying mostly single positive charges [13]. The subsequent times-of-flight for such singly charged proteins along a flight-tube held at high vacuum, following acceleration by means of an electrical field, is proportional to the square root of their mass-over-charge ratios (m/z) [14], from which a characteristic mass spectrum can readily be generated [14]. The mass spectrum of the highly expressed and acid-soluble sub-proteome (including many ribosomal proteins [15]) is generally employed for the characterization/identification of biological samples.

The majority of MALDI-TOF MS sample-preparation methods to date have been developed for use in human clinical microbiology, particularly the diagnosis of bacterial and yeast infections [15]. Common methods have been reviewed in [14], and additional methods have been developed for use with yeasts [16] and fungi [17–20]. Plant-based materials are not, however, particularly well-suited to many of the above methods [21] and so a simplified and relatively inexpensive method for sample preparation that lyses plant cells by biomass-maceration in aqueous acetonitrile containing trifluoroacetic acid (TFA) to selectively extract acid-soluble sample proteins has been developed [22]. This method can also be used with bacteria, fungi, and insects. Cell-lysis and protein-extraction are carried out in near-saturated and less-expensive-grade MALDI matrix, and the resulting lysate, containing matrix and acid-solubilized proteins, is then dried down on the MALDI-TOF MS sample plate and analyzed. This method has previously been used for discrimination between *Impatiens* species and between four UK regional biotypes of *I. glandulifera* that differ in their susceptibility to the biological control agent *P. komarovii* var. *glanduliferae* using leaf material [23].

Extending the scope of MALDI-TOF MS methodology to include seed material, we have recently developed a simple and inexpensive method that generates peak-rich and highly-reproducible MALDI-TOF MS spectra of acid-soluble *I. glandulifera* seed proteins [24] and, using this method, we have been able to discriminate successfully between the same four *I. glandulifera* regional biotypes. This method initially extracts acid-soluble seed proteins in 100 µl of acetonitrile/TFA/water/matrix, followed by further dilution in the same reagent (ideally 20-fold to 100-fold) in order to obtain high-quality spectra. Once again endeavoring to extend the scope of MALDI-TOF MS analysis, we have now incorporated methodology described in Reeve and Buddie [25], who have developed a novel method for the storage of field-sample proteins, which are dried down onto filter paper for subsequent analysis by MALDI-TOF MS. This method was conceived originally in order to overcome one of the most significant limitations of MALDI-TOF MS, namely that relatively-fresh biological material is required, containing proteins that have not yet undergone significant amounts of degradation. This method has the significant advantage that dried proteins, rather than viable plant or seed material (which may be restricted by quarantine regulations for transfer across national borders) can be transported for subsequent MALDI-TOF MS analysis. In the current article, we have successfully adapted the original method of Reeve and Buddie [25] for use with seed material, likewise endeavoring to employ only simple equipment and minimally hazardous reagents prior to sample shipping so as to facilitate field work and method-usage in resource-poor settings.

## Materials and methods

*Impatiens glandulifera* seeds were obtained directly from field populations in 2017. Seed collection was carried out from four sites within the UK: Harmondsworth Moor, Middlesex; Silwood Park, Berkshire; Rhosmaen, Carmarthenshire; and Lampeter, Ceredigion. At each site, seeds were collected from multiple plants. After collection, seeds were air dried at room temperature for 1 week and were then stored in dark at 4°C prior to protein extraction.

Mass spectrometry covering the molecular-weight range from 2 kDa to 20 kDa was carried out using a Bruker Microflex LT linear-mode instrument running the MALDI Biotyper 4.0 applications (Bruker Daltonik, Bremen, Germany) as described in ref. [26].

Single seeds were initially macerated in 500 µl of (50% (v/v) acetonitrile, 2.5% (v/v) TFA, and 47.5% (v/v) water) using the blunt end of a plastic inoculating loop. Seed debris was then pelleted by centrifugation at 14,100 *g* for 1 min in a miniSpin^®^ plus centrifuge (Eppendorf, Stevenage, UK). For the control method, 10 µl of supernatant were mixed with 100 µl of (11 mg/ml α-cyano-4-hydroxycinnamic acid (HCCA) matrix in 65% (v/v) acetonitrile, 2.5% (v/v) TFA, and 32.5% (v/v) water) (referred to as Solution 1 below), and 1 µl was then pipetted onto the Bruker sample plate, air dried, and loaded into the spectrometer. For test-method 1, 10 µl of supernatant were pipetted onto 6-mm-diameter circles of Whatman filter paper, grade 3 (prepared using a hole punch), followed by air drying for 1 h in a fume hood. Immediately or after storage at 35°C as indicated, the dry filter-paper disc was extracted in 100 µl of Solution 1 by immersion and soaking for 20 min followed by vortexing. One microliter of the extraction buffer was then pipetted onto the Bruker sample plate, air dried, and loaded into the spectrometer. For test-method 2, 10 µl of supernatant were pipetted onto 6-mm-diameter circles of Whatman filter paper, grade 3, followed by air drying. Ten microliters of ethanol were then pipetted onto the filter-paper disc, followed by air drying. Immediately or after storage at 35°C as indicated, the dry filter-paper disc was extracted in 100 µl of Solution 1 by immersion and soaking for 20 min followed by vortexing. One microliter of the extraction buffer was then pipetted onto the Bruker sample plate, air dried, and loaded into the spectrometer.

For the test-methods time courses, a single *I. glandulifera* seed from Harmondsworth Moor was macerated and multiple filter-paper discs were prepared using test-methods 1 and 2 as described above. Acid-soluble seed proteins were then extracted and analyzed as described above at *t* = 0 and after 1, 2, 3, and 4 weeks of storage in capped 1.5 ml Eppendorf tubes at 35°C (chosen as a reasonable laboratory model extending to challenging field-storage and shipping conditions with respect to potential protein degradation). A control-method time course was not carried out because of the potential risk of uncapping of tubes containing flammable solvent and volatile acid at 35°C. In order to assess degradation of seed proteins in water over time, a second *I. glandulifera* seed from Harmondsworth Moor was macerated in 500 µl of water using the blunt end of a plastic inoculating loop. Seed debris was then pelleted by centrifugation at 14,100 *g* for 1 min. Ten microliter aliquots of supernatant were placed in capped 1.5 ml Eppendorf tubes and acid-soluble seed proteins were then extracted and analyzed as described above for the control method at *t* = 0 and after 1, 2, 3, and 4 weeks of storage at 35°C. ‘Reference’ sample preparations were carried out as above, from which a database of reference spectra was generated for each method. For spectral comparison, all time-course samples were compared against the database of reference spectra and Bruker identification scores were generated as described in ref. [26].

## Results

**Figure 1:**
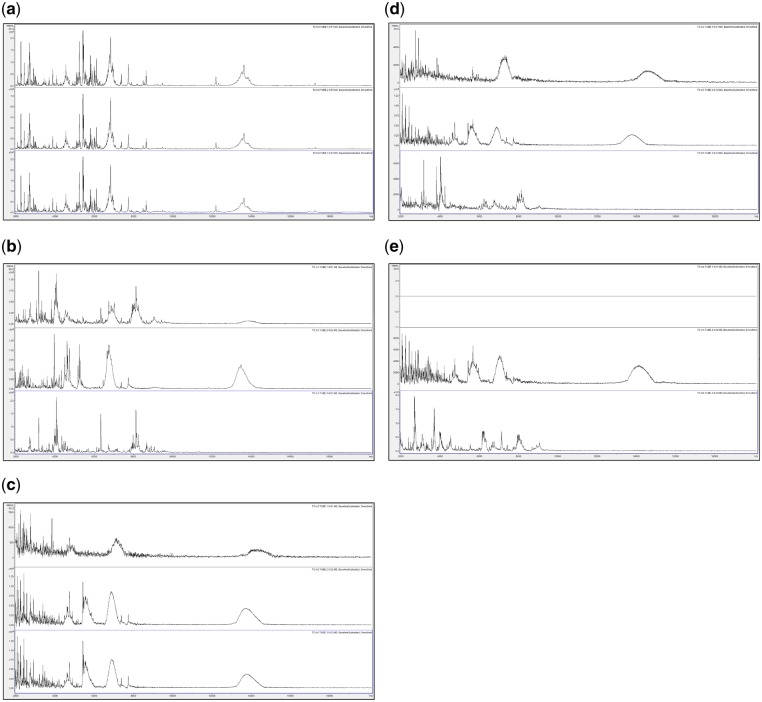
Triplicate acid-soluble seed-protein MALDI-TOF MS spectra from *I. glandulifera* originating from Harmondsworth Moor, obtained from control seed-protein samples stored in water as described in the ‘Materials and methods’ section, (a) at *t* = 0, (b) after 1 week of storage at 35°C, (c) after 2 weeks of storage at 35°C, (d) after 3 weeks of storage at 35°C, and (e) after 4 weeks of storage at 35°C.

**Figure 2:**
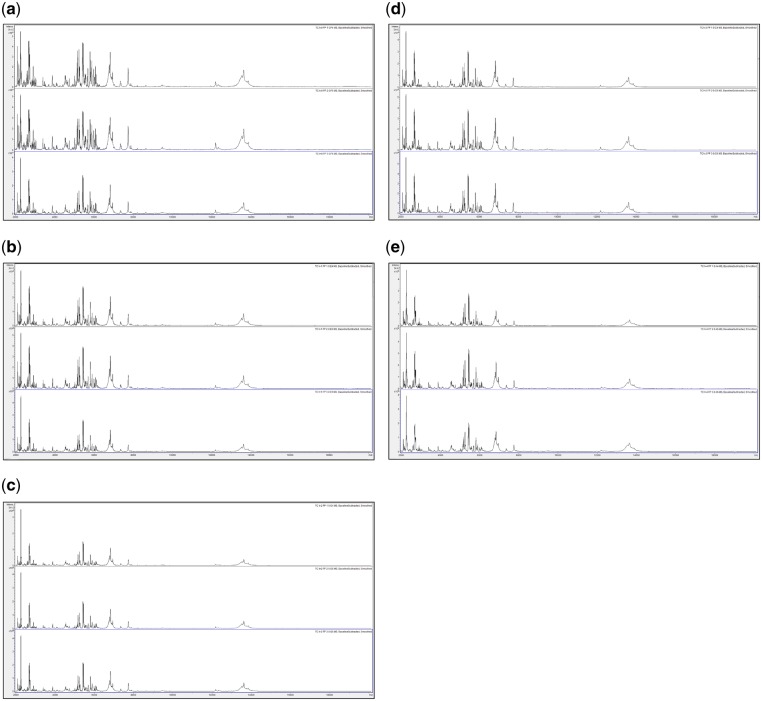
Triplicate acid-soluble seed-protein MALDI-TOF MS spectra from *I. glandulifera* originating from Harmondsworth Moor, obtained using test-method 1 as described in the ‘Materials and methods’ section, (a) at *t* = 0, (b) after 1 week of storage at 35°C, (c) after 2 weeks of storage at 35°C, (d) after 3 weeks of storage at 35°C, and (e) after 4 weeks of storage at 35°C.

**Figure 3:**
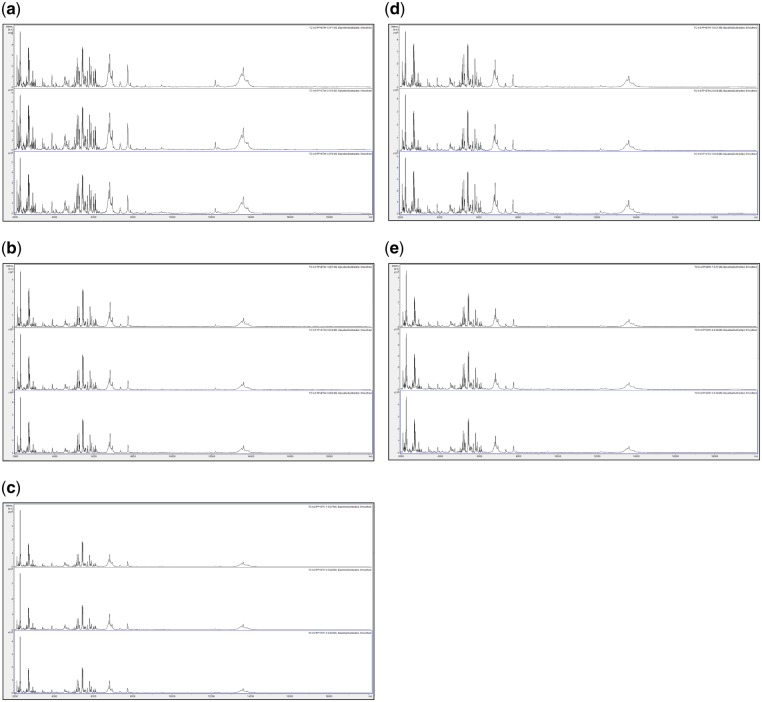
Triplicate acid-soluble seed-protein MALDI-TOF MS spectra from *I. glandulifera* originating from Harmondsworth Moor, obtained using test-method 2 as described in the ‘Materials and methods’ section, (a) at *t* = 0, (b) after 1 week of storage at 35°C, (c) after 2 weeks of storage at 35°C, (d) after 3 weeks of storage at 35°C, and (e) after 4 weeks of storage at 35°C.

In order to assess changes in spectral quality over storage time, Figs 1–3 show triplicate acid-soluble seed-protein MALDI-TOF MS spectra from *I. glandulifera* originating from Harmondsworth Moor, obtained from control seed-protein samples stored in water, and using test-methods 1 and 2 as described in the ‘Materials and methods’ section, at *t* = 0, and after 1, 2, 3, and 4 weeks of storage at 35°C.

Figures 1–3 show that *I. glandulifera* acid-soluble seed-proteins degrade when stored in water (Fig. 1), with visible spectral changes in all three samples after 1 week of storage at 35°C, extensive peak broadening in all three samples from 2 weeks of storage at 35°C, and complete loss of the replicate 1 spectrum after 4 weeks of storage at 35°C. Using both test-methods 1 and 2 (Fig. 2 and Fig. 3, respectively), there is a small reduction in signal strength for the 2-week time points but otherwise the general distribution of peak molecular weights remains constant with storage at 35°C.

**Figure 4:**
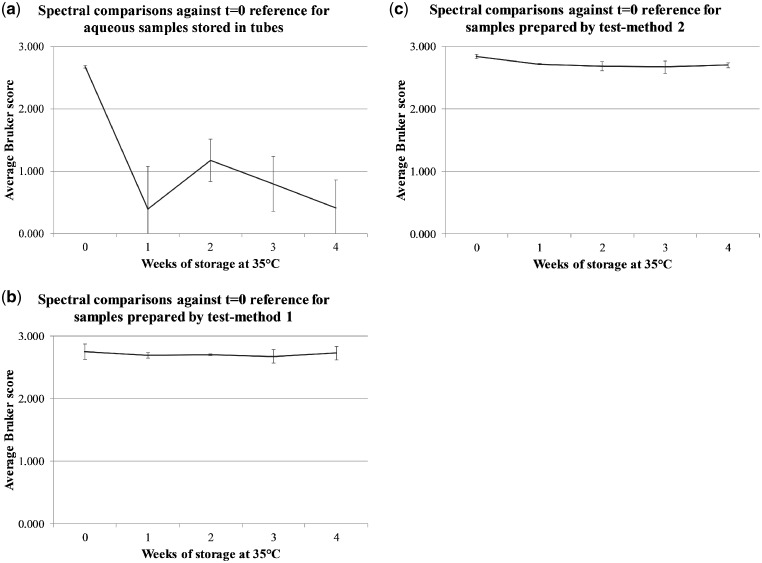
Average Bruker scores, with errors bars indicating one standard deviation either side of the mean, for spectral comparisons between cognate *t* = 0 reference spectra and Harmondsworth Moor *I. glandulifera* acid-soluble seed-protein MALDI-TOF MS spectra, obtained from (a) control seed-protein samples stored in water and using (b) test-method 1, and (c) test-method 2, at *t* = 0, and after 1, 2, 3, and 4 weeks of storage at 35°C.

In order to assess sample degradation over storage time, Figure 4 shows average Bruker scores, with errors bars indicating one standard deviation either side of the mean, for spectral comparisons between cognate *t* = 0 reference spectra and Harmondsworth Moor *I. glandulifera* acid-soluble seed-protein MALDI-TOF MS spectra, obtained from control seed-protein samples stored in water and using test-methods 1 and 2, at t = 0, and after 1, 2, 3, and 4 weeks of storage at 35°C.

Figure 4 shows that *I. glandulifera* acid-soluble seed-proteins degrade when stored in water, with average Bruker scores dropping below 2.0 after storage at 35°C for 1 week. Using test-methods 1 and 2, while the spectra in Figs 1–3 show a reduction in signal strength for some of the time points, the average Bruker scores remain high, and significantly above 2.0, during storage at 35°C for the duration of the time course.

**Figure 5:**
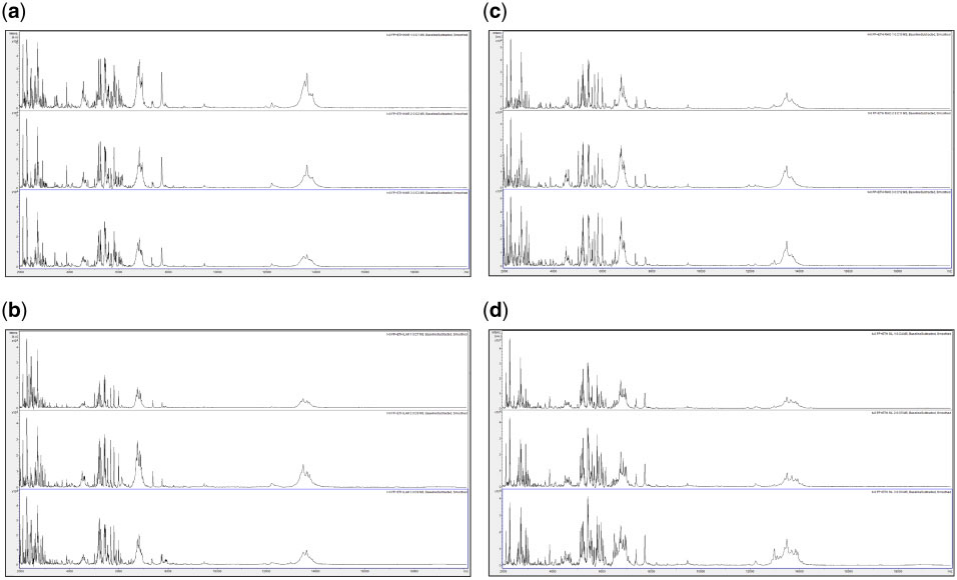
MALDI-TOF MS spectra, obtained using the control method as described in the ‘Materials and Methods’ section, at *t* = 0, of acid-soluble seed proteins from triplicate *I. glandulifera* seeds collected from (a) Harmondsworth Moor, (b) Lampeter, (c) Rhosmaen, and (d) Silwood Park.

**Figure 6:**
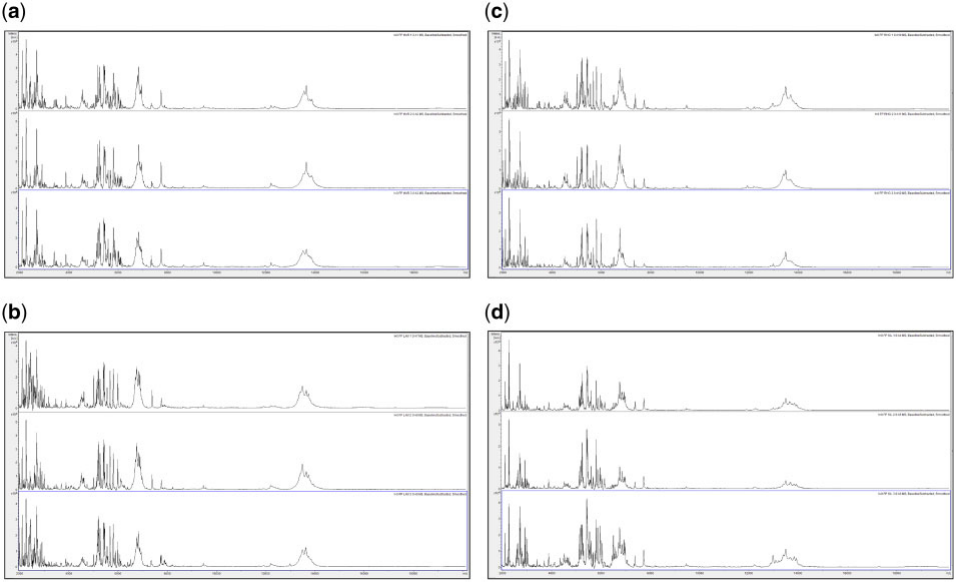
MALDI-TOF MS spectra, obtained using test-method 1, at *t* = 0, of acid-soluble seed proteins from triplicate *I. glandulifera* seeds collected from (a) Harmondsworth Moor, (b) Lampeter, (c) Rhosmaen, and (d) Silwood Park.

**Figure 7:**
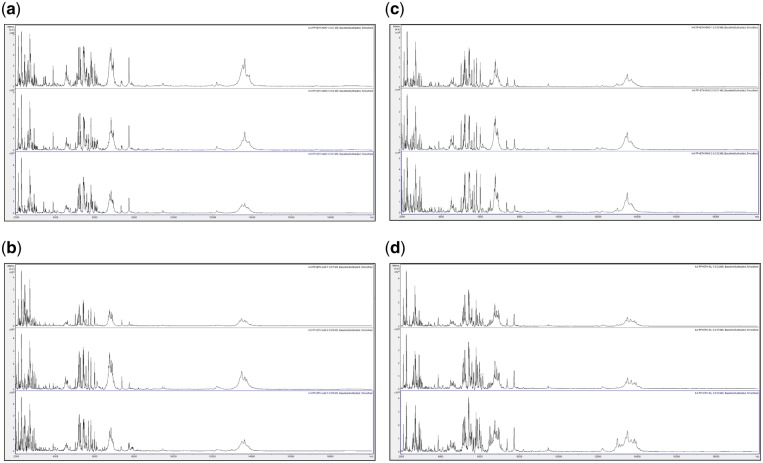
MALDI-TOF MS spectra, obtained using test-method 2, at *t* = 0, of acid-soluble seed proteins from triplicate *I. glandulifera* seeds collected from (a) Harmondsworth Moor, (b) Lampeter, (c) Rhosmaen, and (d) Silwood Park.

**Figure 8:**
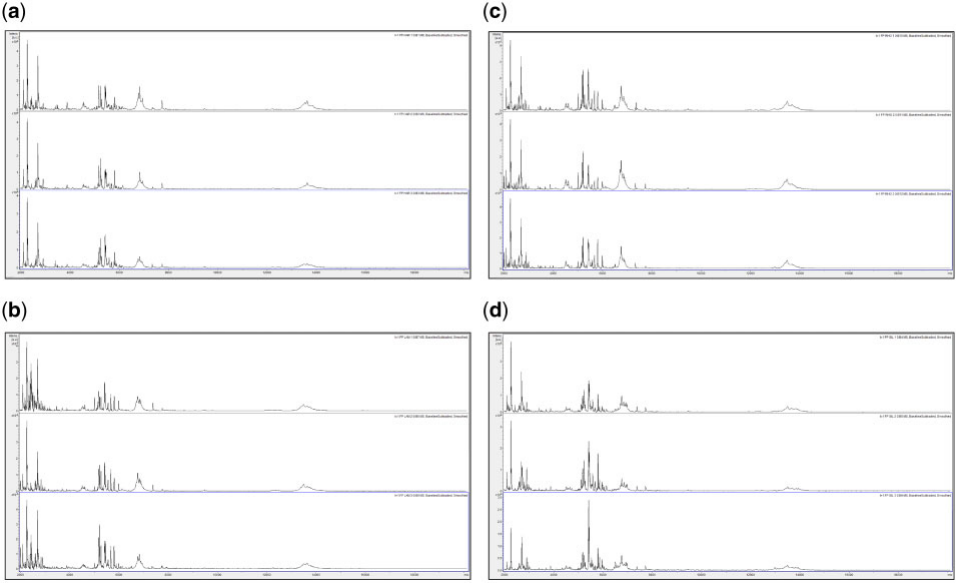
MALDI-TOF MS spectra, obtained using test-method 1, after one month of storage at 35°C, of acid-soluble seed proteins from triplicate *I. glandulifera* seeds collected from (a) Harmondsworth Moor, (b) Lampeter, (c) Rhosmaen, and (d) Silwood Park.

**Figure 9:**
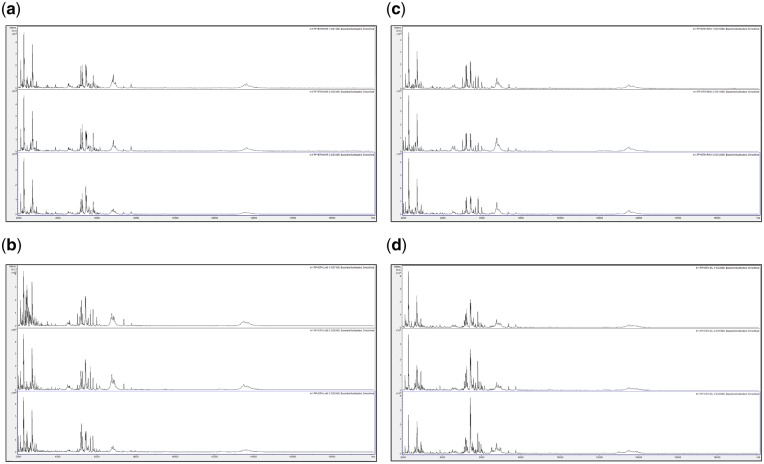
MALDI-TOF MS spectra, obtained using test-method 2, after one month of storage at 35°C, of acid-soluble seed proteins from triplicate *I. glandulifera* seeds collected from (a) Harmondsworth Moor, (b) Lampeter, (c) Rhosmaen, and (d) Silwood Park.

In order to assess spectral change after extended storage at high temperature, Figs 5–9 show MALDI-TOF MS spectra, obtained at *t* = 0 using the control method as described in the ‘Materials and methods’ section, test-method 1, and test-method 2 and MALDI-TOF MS spectra obtained using test-methods 1 and 2 after 1 month of storage at 35°C, of acid-soluble seed proteins from triplicate *I. glandulifera* seeds collected from Harmondsworth Moor, Lampeter, Rhosmaen, and Silwood Park.

Figs 5–9 show that peak-rich and highly reproducible mass spectra are obtained, using the control method, test-method 1, and test-method 2 at *t* = 0, and even after 1 month of storage at 35°C using test-methods 1 and 2, for *I. glandulifera* seeds collected from Harmondsworth Moor, Lampeter, Rhosmaen, and Silwood Park.

**Figure 10:**
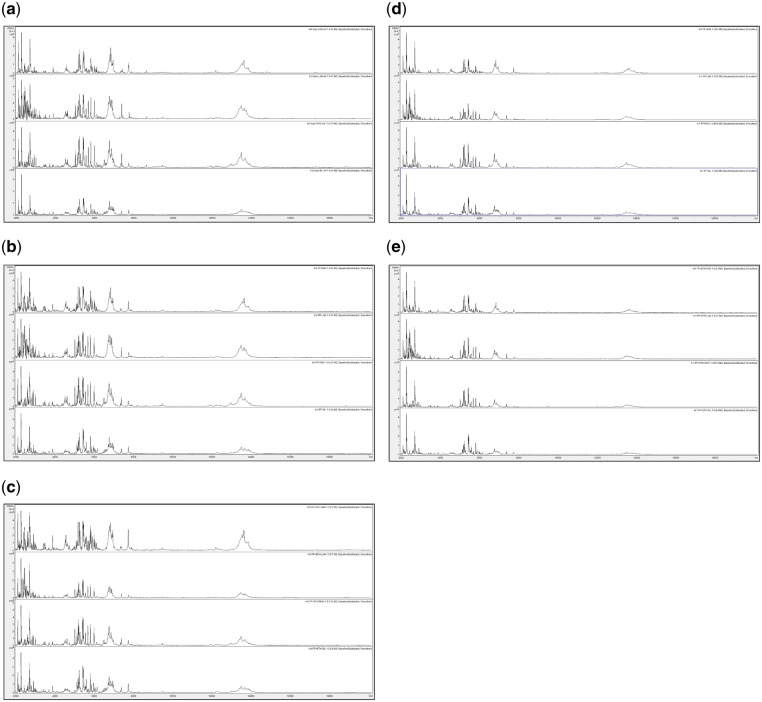
Replicate-1 MALDI-TOF MS spectra of acid-soluble *I. glandulifera* seed proteins from, within each panel from top to bottom, Harmondsworth Moor, Lampeter, Rhosmaen, and Silwood Park, obtained using (a) the control method at *t* = 0, (b) test-method 1 at *t* = 0, (c) test-method 2 at *t* = 0, (d) test-method 1 after 1 month of storage at 35°C, and (e) test-method 2 after 1 month of storage at 35°C.

In order to compare spectra of samples between the different sites before and after extended storage at high temperature, Figure 10 shows (combined into a single figure rather than across multiple figures) comparisons between replicate-1 MALDI-TOF MS spectra of acid-soluble *I. glandulifera* seed proteins from Harmondsworth Moor, Lampeter, Rhosmaen, and Silwood Park, obtained using the control method, test-method 1, and test-method 2 at *t* = 0 and test-methods 1 and 2 after 1 month of storage at 35°C.

Figure 10 shows that peak-rich and reproducible mass spectral comparisons are obtained using the control method, test-method 1, and test-method 2 at *t* = 0, and even after 1 month of storage at 35°C using test-methods 1 and 2, for *I. glandulifera* seeds collected from Harmondsworth Moor, Lampeter, Rhosmaen, and Silwood Park.

**Figure 11:**
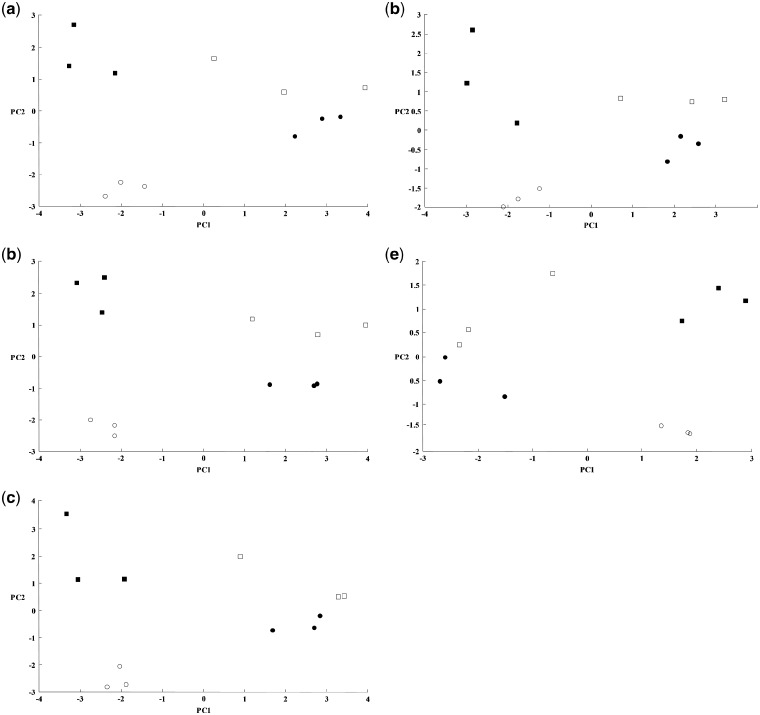
PCA ordination plots from triplicate MALDI-TOF MS spectra of acid-soluble *I. glandulifera* seed proteins from (filled squares) Harmondsworth Moor, (open squares) Lampeter, (filled circles) Rhosmaen, and (open circles) Silwood Park, obtained using (a) the control method at *t* = 0, (b) test-method 1 at *t* = 0, (c) test-method 2 at *t* = 0, (d) test-method 1 after 1 month of storage at 35°C, and test-method 2 after 1 month of storage at 35°C. The components of the first principal component vector (PC1) are shown in the X direction and the components of the orthogonal second principal component vector (PC2) shown in the Y direction to give an ordination plot.

In order to assess discrimination between samples between the different sites before and after extended storage at high temperature, Fig. 11 shows PCA ordination plots from triplicate MALDI-TOF MS spectra of acid-soluble *I. glandulifera* seed proteins from Harmondsworth Moor, Lampeter, Rhosmaen, and Silwood Park, obtained using the control method, test-method 1, and test-method 2 at *t* = 0, and test-methods 1 and 2 after 1 month of storage at 35°C.

Figure 11 shows that, for all methods used, and before and after storage for 1 month at 35°C, the spectra from the samples originating from Harmondsworth Moor (filled squares) and the spectra from the samples originating from Silwood Park (open circles) form clusters on the ordination plot with clear separation from each other and from the remaining sample spectra. The spectra from the samples originating from Rhosmaen (filled circles) and Lampeter (open squares) are closely related to each other, and cannot readily be discriminated objectively by visual inspection of the ordination plots.

## Discussion

Invasive weeds are an economic problem of global significance, for which biological control employing natural enemies isolated from the target-species region of origin can offer an environmentally sustainable and effective management solution. Experience from past weed-biocontrol programs suggests that the susceptibility of target-plant populations to the biological control agent can vary [27, 28]. Current practice for optimizing the pairing between the biological control agent and target-weed regional (or other) biotypes is largely empirical, which can be a slow (and also relatively expensive) process. As an alternative to this approach, we have investigated MALDI-TOF MS as a means of discriminating between regional biotypes with known biological control agent susceptibility profiles because this method is both rapid and very inexpensive in terms of reagent usage and time required for sample processing.

In the current article, we have employed a MALDI-TOF MS method based upon sample preparation that uses acetonitrile containing TFA to selectively extract acid-soluble proteins, with protein-extraction being also carried out in the presence of near-saturated and inexpensive-grade MALDI matrix [22]. In order to test the discriminating power of the method developed in this article, we again chose to work with seeds from four UK regional biotypes of *I. glandulifera* that we have previously demonstrated discrimination between using leaf samples [23] and seed material [24].

The primary motivation behind the continued method development reported in the current article was to facilitate field work and method-usage in resource-poor settings and, to this end, we sought to incorporate methodology described in Reeve and Buddie [25], who have developed a simple and inexpensive method for the practical storage of field-sample proteins, dried down onto filter paper, for subsequent MALDI-TOF MS analysis. This method was conceived in order to overcome one of the most significant limitations of MALDI-TOF MS, namely the requirement for relatively-fresh biological material, containing proteins that have not yet undergone significant degradation, and was originally demonstrated using leaf biomass. In the current article, we have successfully adapted the method of Reeve and Buddie [25] for use with seed material, endeavoring throughout to employ only simple equipment and minimally hazardous reagents prior to sample shipping, consistent with our aim of facilitating field work and method-usage in resource-poor settings. To this end, we employed pre-formulated [50% (v/v) acetonitrile, 2.5% (v/v) TFA, and 47.5% (v/v) water] solution, which is readily available from Sigma Aldrich, Gillingham, UK, in order to reduce the potential hazard from handling undiluted TFA.

In terms of stability over time, we have shown that *I. glandulifera* acid-soluble seed-proteins degrade when stored in water. Using test-methods 1 and 2, in contrast, while there is a reduction in signal strength for some of the time points, average Bruker scores remain high, and significantly above 2.0, during storage at 35°C for the duration of the time course. The cause of this signal-strength reduction is not clear at the moment. Sample degradation seems unlikely as the average Bruker scores do not drop over the time course. It is possible that, with increased time of storage for protein dried down on the filter paper, the efficiency of the extraction process decreases and less protein is recovered after longer storage times, a possibility that we intend to investigate in future work. The observed signal-strength reduction (albeit with no loss of sample utility as judged by the average Bruker scores obtained) can be offset by the advantages that our method affords in allowing the shipment, prior to MALDI-TOF MS analysis, of dried protein, rather than viable seed material (which may be subject to quarantine restriction for transfer across national borders), and removal of any need for freezing or chilling immediately after sampling and keeping samples in this manner between field-work sites and the laboratory.

In terms of distinguishing between regional biotypes of *I. glandulifera*, we have shown that peak-rich and highly reproducible mass spectra are obtained even after 1 month of storage at 35°C using test-methods 1 and 2, for seeds collected from Harmondsworth Moor, Lampeter, Rhosmaen, and Silwood Park. Using these spectra as the basis for PCA ordination plots, we have shown that, for all methods used, and before and after storage for 1 month at 35°C, the spectra from the samples originating from Harmondsworth Moor and the spectra from the samples originating from Silwood Park form clusters with clear separation from each other and from the remaining sample spectra, consistent with previous work [23, 24]. The spectra from the samples originating from Rhosmaen and Lampeter, again consistent with previous work, are observed to be closely related to each other.

In conclusion, we have successfully developed a further MALDI-TOF MS-based method that can generate peak-rich and very-reproducible MALDI-TOF MS spectra from acid-soluble seed proteins, dried down onto filter paper in a manner that retains sample integrity over extended time and/or at elevated temperatures. Dried seed-protein samples immobilized on filter paper remain stable for MALDI-TOF MS analysis, even after 1 month of storage at 35°C. This method may provide a further useful tool for the matching of biological control agents to susceptible (regional) target-plant biotypes, and for seed characterization and/or identification in general.
